# Third Molar Displacement into Submandibular Space

**DOI:** 10.1155/2019/6137868

**Published:** 2019-09-17

**Authors:** Mirlany Mendes Maciel Oliveira, Rodrigo da Franca Acioly, Dennis Dinelly de Souza, Bruno Araújo da Silva, Daniel Do Carmo Carvalho

**Affiliations:** ^1^Bucomaxilofacial Surgery and Traumatology Clinical Hospital of the Federal University of Uberlândia (HC-UFU), Uberlândia, Minas Gerais, Brazil; ^2^Buccomaxillofacial Surgery, Department of Buccomaxillofacial Surgery and Traumatology, Hospital Geral de Roraima, Boa Vista, RR, Brazil

## Abstract

There are various accidents and complications that may occur during extraction of dental elements. The displacement of dental elements to other facial spaces is one kind of the possible complications, and there may be significant physical and psychological results for the patient. The treatment for this kind of occurrence may vary from a conservative technique to surgical procedure, what will depend on clinical characteristics, symptoms, the location of the dental element, and its relation to adjacent structures. The objective of this article is to report a clinical case of the displacement of a lower third molar tooth into the submandibular space during its extraction, followed by surgical removal through extraoral approach, with proservation for the next two years when patient evolved to paresthesia of the inferior alveolar nerve.

## 1. Introduction

The extraction of dental elements is one of the most common procedures in the dental surgeons' routine, either for a general dentist or an oral and maxillofacial surgeon. However, as all other procedures in the dental field, the simplest oral surgeries can also present occurrence of accidents and complications [1].

Several accidents during dental extractions can occur, which include fractured mandible, damage to nervous structures, and displacement of the element into facial spaces [2].

The displacement of teeth to neighboring spaces is a rare intraoperative accident in dental surgery and it can cause physical and psychological damage to the patient [1]. The possible causes of teeth displacement may be associated with excessive pressure, lack of experience of the professional, inadequate use of the surgical equipment, and anatomic characteristics of the patient [2, 3].

Lingually located teeth or deeply impacted molars present a higher risk of displacement to other facial spaces [4]. According to Aznar-Arasa et al. [3], the most common location of displacement is into the maxillary sinus. Other places include submandibular space, lingual space, infratemporal fossa, oral space, pterygomandibular space, and lateral pharyngeal space [2, 5].

The symptoms after teeth displacement into facial spaces may vary from asymptomatic cases to pain report, edema, and mandible trismus, and based on these findings, it is indicated a choice between the removal of the dental element and a conservative treatment [3].

The open surgical technique, together with appropriate use of pressure and placement of retractors of the tongue region, can help minimize the occurrence of this kind of complication [4, 6, 7].

The present article is aimed at reporting a clinical case of the transoperative displacement of a lower third molar tooth into the submandibular space.

## 2. Case Report

**Figure 1:**
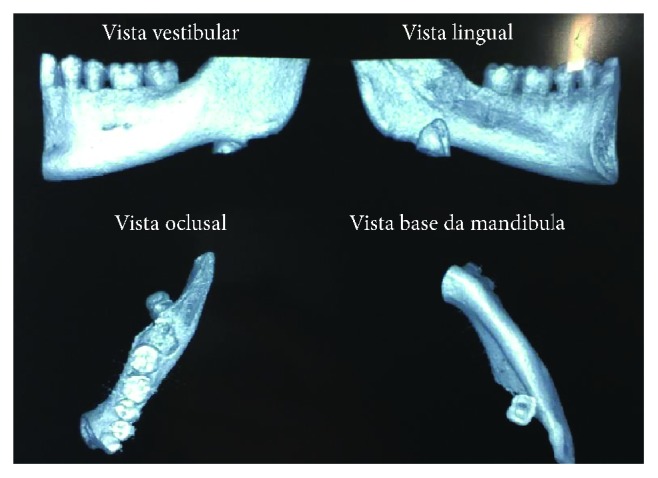
Computed tomography 3D reconstruction.

**Figure 2:**
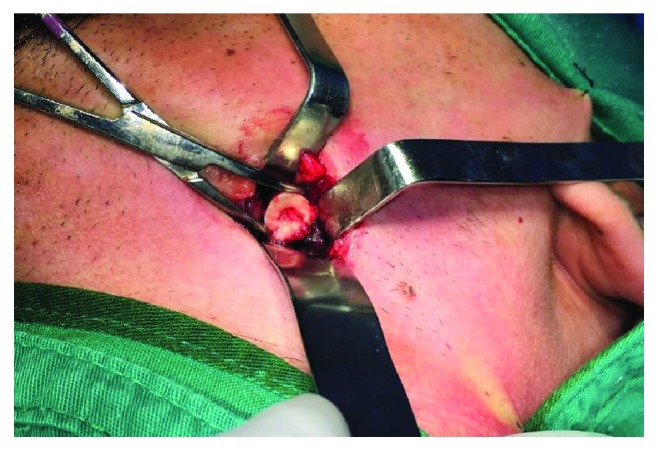
Removal of dental element by extraoral access.

**Figure 3:**
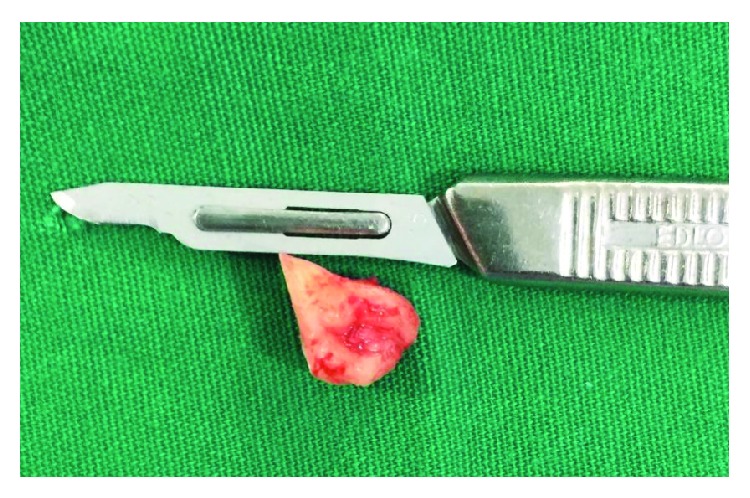
Third molar removed.

Male patient, 21 years old, with leucoderma, denied underlying diseases or allergies, attended to the Oral and Maxillofacial Surgical and Trauma Service of the General Hospital of Roraima-HGRR, with the main symptomatology of pain and edema after an attempt of extraction of the left lower third molar which evolved with complication transoperative of displacement to adjacent spaces. The patient reported that during the attempt to extract the dental element, he felt symptoms of intense pain and he was communicated of the transoperative accident. After the displacement of the dental element, the surgeon dentist attempted the removal of the tooth by using specially one of the fingers, but without success. After image examination of a cone beam computed tomography, the diagnosis of displacement of the third molar into submandibular space was confirmed. The management adopted was the procedure to remove the dental element under general anesthesia, and through an extraoral access on the left submandibular region, an incision in layers was made and the dental element was completely removed. The surgical procedure was performed without further complications, and there was a review of hemostasis, suture of layers using thread vicryl 3-0, external suture using nylon thread 5-0, and review of the systems (Figures 1–3).

After 2 years of proservation, the patient presents preserved masticatory functions, satisfactory mouth opening, and the occurrence of alveolar inferior nerve paresthesia.

## 3. Discussion

Nowadays, the extraction of dental elements is a routine procedure in dental offices. However, individual analysis of each case is needed to perform this procedure.

The surgeon should not underestimate the procedure by considering it easy at first; there should be an efficient preoperative analysis, and after the extraction, a clinical and radiographic analysis of the place the tooth element has been removed from [6, 8].

Surgeon dentists who perform third molar extractions should consider the local characteristics of the dental element in order to evaluate the level of difficulty the surgical procedure might present. These characteristics include level of impactation, root format, dental inclination, and bone density [4, 9].

Bimanual examination can help finding the fragment location, associated to radiograph and tomography images, seeking for the exact local, especially in cases of lower tooth displacement. The advantages of external pressure include avoiding displacement of the fragment, elevation of the mouth floor, and palpation of the area. However, this approach is not recommended if patients present edema or obesity [1]. In case displacement of a dental element into facial spaces occurs, careful thinking should indicate the management procedures to be adopted. Attempts of immediate removal with lack of skills or lack of anatomic and surgical knowledge may worsen the condition by deepening the fragment or moving it into adjacent spaces [5].

If fragments are bigger than 5 mm, there should be a surgical procedure, but if fragment is smaller than 5 mm and it is not palpable, the conservative treatment can be an option. However, if dental element is left for a long period of time, it is possible that there will be reaction to a foreign body as well as the possibility of infection in the neck spaces [4].

The surgeon experience is an important aspect to be considered, but it is not determinant for the occurrence of teeth displacement into facial spaces, because both general surgeon dentists and specialists in Oral and Maxillofacial Surgery may face the occurrence of this kind of accident.

In the international literature, it is possible to find other accidents related to dental removal which include the displacement of a dental high-speed piece bur into submandibular space and a broken needle dislodged into the prevertebral space [5, 10, 11].

## 4. Conclusion

The displacement of dental elements is a rare accident, but potentially serious. Although third molars are the dental elements mostly involved in displacement into facial spaces, the other teeth can also be responsible for these accidents. Thus, the case should be sent to an Oral and Maxillofacial surgeon to provide adequate diagnosis, verify the exact location of the dental fragment through imaging exams, and plan the removal or not of the element, based on clinical characteristics, location, noble adjacent structures, and size of the element located in the neighboring facial spaces.

## References

[1] Zhao S., Huang Z., Geng T., Huang L. (2015). Intraoral management of iatrogenically displaced lower third molar roots in the sublingual space: a report of 2 cases. *International Journal of Clinical and Experimental Medicine*.

[2] Chang W., Chang T., Chiu K., Wu C., Chen Y. (2014). Accidental iatrogenic displacement of the mandibular third molar into the lateral pharyngeal space-a case report. *Tawian J Oral Maxillofac Surg*.

[3] Aznar-Arasa L., Figueiredo R., Gay-Escoda C. (2012). Iatrogenic displacement of lower third molar roots into the sublingual space: report of 6 cases. *Journal of Oral and Maxillofacial Surgery*.

[4] Nusrath M. A., Banks R. J. (2010). Unrecognised displacement of mandibular molar root into the submandibular space. *British Dental Journal*.

[5] Yalcin S., Aktas I., Emes Y., Atalay B. (2008). Accidental displacement of a high-speed handpiece bur during mandibular third molar surgery: a case report. *Oral Surgery, Oral Medicine, Oral Pathology, Oral Radiology, and Endodontics*.

[6] Bozkurt P., Erdem E. (2017). Management of upper and lower molars that are displaced into the neighbouring spaces. *British Journal of Oral and Maxillofacial Surgery*.

[7] Campbell A., Costello B. J. (2010). Retrieval of a displaced third molar using navigation and active image guidance. *Journal of Oral and Maxillofacial Surgery*.

[8] Medeiros N., Gaffrée G. (2008). Accidental displacement of inferior third molar into the lateral pharyngeal space: case report. *Journal of Oral and Maxillofacial Surgery*.

[9] Sverzut C. E., Trivellato A. E., Sverzut A. T., Matos F. P., Kato R. B. (2009). Removal of a maxillary third molar accidentally displaced into the infratemporal fossa via intraoral approach under local anesthesia: report of a case. *Journal of Oral and Maxillofacial Surgery*.

[10] Kamburoglu K., Kursun S., Oztas B. (2010). Submandibular displacement of a mandibular third molar root during extraction: a case report. *Cases Journal*.

[11] Sahin B., Yildirimturk S., Sirin Y., Basaran B. (2017). Displacement of a broken dental injection needle into the perivertebral space. *Journal of Craniofacial Surgery*.

